# Time-Domain Data Fusion Using Weighted Evidence and Dempster–Shafer Combination Rule: Application in Object Classification

**DOI:** 10.3390/s19235187

**Published:** 2019-11-26

**Authors:** Md Nazmuzzaman Khan, Sohel Anwar

**Affiliations:** Department of Mechanical and Energy Engineering, Indiana University—Purdue University Indianapolis, Indianapolis, IN 46224, USA; soanwar@iupui.edu

**Keywords:** evidence combination, time-domain data fusion, object classification, uncertainty

## Abstract

To apply data fusion in time-domain based on Dempster–Shafer (DS) combination rule, an 8-step algorithm with novel entropy function is proposed. The 8-step algorithm is applied to time-domain to achieve the sequential combination of time-domain data. Simulation results showed that this method is successful in capturing the changes (dynamic behavior) in time-domain object classification. This method also showed better anti-disturbing ability and transition property compared to other methods available in the literature. As an example, a convolution neural network (CNN) is trained to classify three different types of weeds. Precision and recall from confusion matrix of the CNN are used to update basic probability assignment (BPA) which captures the classification uncertainty. Real data of classified weeds from a single sensor is used test time-domain data fusion. The proposed method is successful in filtering noise (reduce sudden changes—smoother curves) and fusing conflicting information from the video feed. Performance of the algorithm can be adjusted between robustness and fast-response using a tuning parameter which is number of time-steps($t_{s}$).

## 1. Introduction

Dempster–Shafer theory (DS theory), also called belief function theory, as introduced and developed by Dempster and Shafer [1,2], has emerged from their works on statistical inference and uncertain reasoning. As a tool to manipulate an uncertain environment, DS evidence theory established a rounded system for uncertainty management and information fusion [3,4,5,6]. The research is mainly focused on multi-sensor fusion in space-domain where multiple pieces of evidence are gathered from multiple sensors and combined to achieve a decision-level fusion. However, for real-time application of multi-sensor systems, time-domain evidence fusion is also needed. Due to noise and disturbances from environment or wrong output from sensors in space-domain; noisy, distorted or even wrong results can be obtained at a certain time-step. The goal of the time-domain evidence fusion is, by using the information available at previous time-steps, to capture the dynamic behavior of the system and reduce the disturbance of the final output.

Few studies considered the influence of time factor on time-domain evidence combination. Hong and Lynch [7] showed multiple approaches of how original DS method can be applied to time-domain, but no steps to improve the limitations of the original DS method [8] is mentioned. Song et al. [9,10] proposed credibility decay model based on the idea that credibility of the evidence will decay over time. However, his methods showed poor anti-disturbing ability when conflicting (noisy) evidence is present in time-domain. Chengkun et al. [11] proposed an improved credibility decay model using exponential smoothing and conflict degree between pieces of evidence. His method showed relatively better anti-disturbing ability compared to Song’s methods, but the convergence rate was poor. Moreover, no research showed the effect of their algorithm on real-time noisy data.

In recent times, convolution neural network (CNN) has been successfully used to classify weed vs. crops, multiple types of weeds or crops [12,13,14]. All the works found in the literature classify weed or crop from input image. Also, no work included the uncertainties inherent to the CNN-based classifiers into the classification output. DS framework is an effective method to include uncertainties into classification output. Construction of basic probability assignment (BPA) based on confusion matrix to include uncertainties is a practical method [15,16]. However, how these uncertainties affect the real-time classification from video feed under DS framework is still unknown.

**Figure 1:**
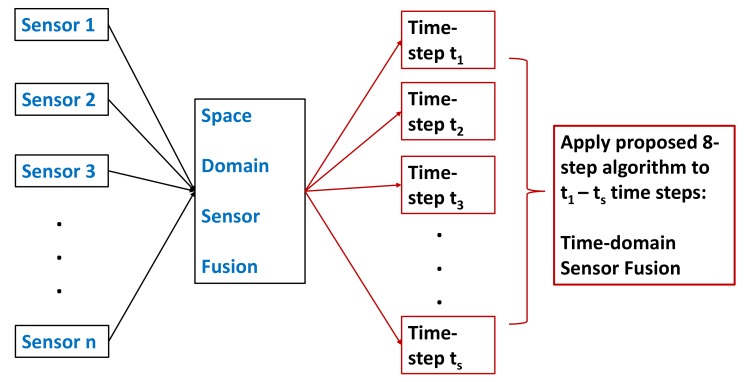
Simple representation of sensor fusion in space and time-domain.

In this research an evidence-fusion algorithm is proposed which can be applied to both space and time-domain (application in time-domain is presented in this paper). Multiple detailed example showed how this algorithm performed under noisy or erroneous data. This algorithm is also tested with real-time classification data. A CNN is trained to classify three different objects. Real-time classification from video feed is used for time-domain data fusion. To incorporate CNN’s classification uncertainty, precision and recall values from the confusion matrix is included into the BPA. Results showed, number of time-steps ($t_{s}$) is a tuning parameter. Based on $t_{s}$, time-domain fused results could be robustness oriented or fast-response oriented. Figure 1 shows a simple representation of where this algorithm can be applied.

## 2. Dempster–Shafer Evidence-Based Combination Rule

### 2.1. Frame of Discernment (FOD)

The frame of discernment contains M mutually exclusive and exhaustive events (also represented by X in this research).

(1) $$X=\Theta =\{\theta_{1},\theta_{2}\ldots ,\theta_{M}\}$$

The representation of uncertainties in the DS theory is similar to that in conventional probability theory and involves assigning probabilities to the space Θ. However, the D-S theory has one significant new feature: it allows the probability to be assigned to subsets of Θ as well as the individual element $\theta_{i}$. Accordingly, we can derive the power set $2^{\Theta }$ of DS theory:

(2) $$2^{\Theta }=\left\{\phi ,\left\{\theta_{1}\right\},\left\{\theta_{2}\right\},\ldots ,\left\{\theta_{1},\theta_{M}\right\},\ldots ,\Theta \right\}$$

where ϕ is empty set. It is clearly seen in (2) that the power set $2^{\Theta }$ has $2^{M}$ propositions. Any subset except singleton of possible values means their union. For example, $\left\{\theta_{1},\theta_{2},\theta_{3}\right\}\equiv \left\{\theta_{1}\cup \theta_{2}\cup \theta_{3}\right\}$. Complete probability assignment to power set is called BPA.

### 2.2. Basic Probability Assignment (BPA) / Mass Function

Evidence in DS theory is acquired by multi-sensor information. Mass function (mass) is a function, $m:2^{\Theta }\to \left[0,1\right]$ that satisfies (3) and (4):

(3) m(ϕ)=0

(4) $$\sum_{\theta \in 2^{\theta }}m(\theta )$$

m is called basic probability assignment. Elements of power set with m(θ)>0 is called focal elements. This can be explained with the help of a simple example. Let the three objects to be detected be, Θ={a,b,c}. Powerset, $2^{\Theta }=2^{3}=\left\{\phi ,a,b,c,\left\{a,b\right\},\left\{a,c\right\},\left\{b,c\right\},\Theta \right\}$. From a sensor or by an expert following mass values are assigned, m(a)=0.2,m(b)=0.3,m(a,b)=0.4,m(a,b,c)=0.1. The four subsets are called focal elements.

### 2.3. Dempster–Shafer Rule of Combination

The purpose of data fusion is to summarize and simplify information rationally obtained from independent and multiple source. It emphasizes on the agreement between multiple sources and ignores all the conflicting evidence through normalization. the DS combination rule for combining two pieces of evidence $m_{1}$ and $m_{2}$ is defined:

(5) $$m_{12}\left(A\right)=\frac{\sum_{B\cap C}\left\{m_{1}\left(B\right)\cdot m_{2}\left(C\right)\right\}}{1-K}$$

when A≠ϕ and m(ϕ)=0.

(6) $$K=\sum_{B\cap C=\phi }\left\{m_{1}\left(B\right)\cdot m_{2}\left(C\right)\right\}$$

where *K* is the degree of conflict in two sources of evidence. The denominator (1−*K*) is a normalization factor, which helps aggregation by completely ignoring the conflicting evidence and is calculated by adding up the products of BPA’s of all sets where intersection is null. DS combination rule in (5) conforms to both commutative and associate law.

$$m_{1}⊕m_{2}=m_{2}⊕m_{1}$$

$$\left(m_{1}⊕m_{2}\right)⊕m_{3}=m_{1}⊕\left(m_{2}⊕m_{3}\right)$$

### 2.4. Belief and Plausibility Function

Given a basic assignment m we can define a belief function: Bel: $m:2^{\Theta }\to \left[0,1\right]$, such that for any A⊂Θ:

(7) $$Bel\left(A\right)=\sum_{B\subseteq A}\left\{m\left(B\right)\right\}$$

Bel (A) measures the belief that the element is member of A. m(A) measures the amount of belief that one commits exactly to A alone, Bel(A) measures the total belief that the special element is in A. Based on the same premise,

(8) $$Pl\left(A\right)=1-Bel\left(\bar{A}\right)$$

Pl(A) measures the degree to which one fails to doubt A. Pl(A) measures the total belief mass that can move into A, whereas Bel(A) measures the total belief mass that is constrained to A.

### 2.5. Entropy under DS Framework

Information is a function of distribution. Entropy measures the compactness of a distribution of information. Information is a measure of the compactness of a distribution; logically if a probability distribution is spread evenly across many states, then its information content is low, and conversely, if a probability distribution is highly peaked on a few states, then its information content is high [17]. The proposed entropy function is based on Shannon [18] and Deng [19] entropy, which considers Bel and Pl of mass function, cardinality of focal elements and number of elements in FOD.

(9) $$Shannon\;Entropy,E_{Sh}=-\sum_{i=1}^{n}p_{i}.log_{2}\left(p_{i}\right)$$

(10) $$Deng\;Entropy,E_{Deng}=-\sum m\left(A\right).log_{2}\frac{m(A)}{\left(2^{\left|A\right|}-1\right)}$$

(11) $$Proposed\;Entropy,E_{p}=-\sum \frac{Bel(A)+Pl(A)}{2}\cdot \;log_{2}\left(\frac{Bel(A)+Pl(A)}{2.(2^{\left|A\right|}-1)}\cdot \;\exp^{\left(\frac{|A|-1}{\left|X\right|}\right)}\right)$$

where n is the amount of basic states in a state space, $p_{i}$ is the probability of state *i*, |*A*| denotes the cardinality of the focal element *A*, |*X*| represents the number of elements in FOD. Following example can show how the proposed entropy function captures entropy under DS framework.

**Example** **1.**  *In a target identification problem, two reliable sensors report the results independently. The results are represented by BPA as follows:*

$Sensor\;1:m_{1}:m_{1}\left(a,b\right)=0.4,m_{1}\left(c,d\right)=0.6$

$Sensor\;2:m_{2}:m_{2}\left(a,c\right)=0.4,m_{2}\left(b,c\right)=0.6$

*If we calculate Shannon and Deng entropy of the sensors we get the following:*

*Shannon Entropy: $m_{1}=0.971,m_{2}=0.971$ and Deng Entropy: $m_{1}=2.55,m_{2}=2.55$.*

*First, sensor 1 is classifying between 4 objects and sensor 2 is classifying between 3 objects. Sensor 1 should have higher uncertainty and entropy in this regard. Secondly, focal elements of sensor 1 are peaked between two separate states compared to sensor 2. In sensor 2, state c is common between two focal elements and it creates more uncertainty. Shannon and Deng entropy failed to capture these uncertainties and as a result they have equal uncertainties for both sensors. The proposed entropy function captures the uncertainties in two steps. The exponential factor*$\exp^{\left(\frac{|A|-1}{\left|X\right|}\right)}$*in the new belief entropy represents the uncertain information in number of elements of FOD. The lower and upper bounds of evidence* ($\frac{Bel(A)+Pl(A)}{2}$), *captures the uncertainty when states are shared between focal elements. As a result, the following entropy can be calculated using the proposed entropy function:*

*Proposed Entropy:*$m_{1}=2.195,m_{2}=2.27$. *A separate detailed work on this novel entropy function and its properties can be found in the literature [20].*

## 3. Proposed Algorithm for Time-Domain Data Fusion

The proposed method is a distance-based method. It calculates the relative distances between the sensor data at each time-step (classification output). Then based on average distance, it classifies which time-step output is credible and which time-step output is incredible. Then it penalizes the incredible time-step output using the entropy function so that incredible time-step has less effect on fused output. It also rewards the credible time-step input so that credible time-step carries more weight towards fused output. Credible time-step is the time-step which contains credible or true data. Incredible time-step contains untrue or unreliable data. At the end, modified evidence is fused using original DS sensor fusion equation. The proposed algorithm can also be applied to space-domain sensor fusion with some minor modification [20]. As Figure 1 suggests, in space-domain, multiple physical sensors are used to collect data. As an example, each sensor could be a camera. From each camera, multiple object classification can be obtained when video feed goes through a neural network-type classifier. Classification ID’s from multiple cameras can be fused together using the proposed algorithm. Due to faulty sensors or obstructed view, neural network output from cameras can generate wrong classification ID. In space-domain, the proposed algorithm finds out which sensors are generating wrong classification ID and penalizes the wrong classification ID by assigning less weight to that sensor output.

**Step 1**: Build a multi-time-steps information matrix. Assume there are N time-steps in the frame $\Theta =\{H_{1},H_{2},\ldots ..,H_{M}\}$.

(12) $$\left(\begin{array}{cccc} m_{1}\left(H_{1}\right) & m_{1}\left(H_{2}\right) & \cdots & m_{1}\left(H_{M}\right)\\ m_{2}\left(H_{1}\right) & m_{2}\left(H_{2}\right) & \cdots & m_{2}\left(H_{M}\right)\\ \vdots & \vdots & \ddots & \vdots \\ m_{N}\left(H_{1}\right) & m_{N}\left(H_{2}\right) & \cdots & m_{N}\left(H_{M}\right) \end{array}\right)=\left(\begin{array}{c} t_{1}\\ t_{2}\\ \vdots \\ t_{s} \end{array}\right)$$

**Step 2**: Measure the relative distance between each time-step data. Several distance function can be used to measure the relative distance. They all have their own advantages and disadvantages regarding runtime and accuracy. We have used Jousselme’s distance [21] function. Jousselme’s distance function uses cardinality in measuring distance which is an important metric when multiple elements are present in one BPA under DS framework. Assuming that there are two mass functions indicated by $m_{i}$ and $m_{j}$ on the discriminant frame Θ, the Jousselme distance between $m_{i}$ and $m_{j}$ is defined as:

(13) $$DM\left(m_{i},m_{j}\right)\;=\;\sqrt{\frac{1}{2}\cdot \left(m_{i}-m_{j}\right)\cdot D\cdot {\left(m_{i}-m_{j}\right)}^{T}}$$

where $D=\frac{\left|A\cap B\right|}{\left|A\cup B\right|}$, and |.| represents cardinality.

**Step 3**: Calculate sum of distance for each time-step.

(14) $$d_{i}\;=\;\sum_{j=1\;\&\;j\neq i}^{N}DM\left(m_{i},m_{j}\right)$$

**Step 4**: Calculate global average of distance for all time-steps considered.

(15) $$\bar{d}\;=\;\frac{\sum_{i=1}^{N}d_{i}}{N}$$

**Step 5**: Calculate belief entropy for each time-step using (11) and normalize:

(16) $$\bar{E_{P}\left(m_{i}\right)}\;=\;\frac{E_{p}\left(m_{i}\right)}{\sum E_{p}\left(m_{i}\right)}$$

**Step 6**: The time-step data set is divided into two parts: the credible time-step and the incredible time-step.

(17) $$\begin{array}{c} If\;d_{i}\leq \bar{d},m_{i}\;is\;credible\;time\;step\\ If\;d_{i}>\bar{d},m_{i}\;is\;incredible\;time\;step \end{array}$$

The intuition is that if data at a specific time-step has higher distance than average distance then probably that time-step is faulty and should be penalized (incredible time-step). If data at a specific time-step is lower than average, then that time-step is in harmony with other time-steps and should be rewarded (credible time-step). As a result, following Reward and Penalty function is proposed:

(18) $$\begin{array}{c} For\;credible\;time\;step,\;Reward\;function\;=-ln(\bar{E_{P}\left(m\right)})\\ For\;incredible\;time\;step,\;Penalty\;function\;=-ln(1-\bar{E_{P}\left(m\right)}) \end{array}$$

Reward and penalty values are normalized to get the weight of each time-step.

(19) $$\bar{w_{i}}\;=\;\frac{Reward\;or\;Penalty}{\sum Reward\;or\;Penalty}$$

**Step 7**: Modify the original data of each time-step.

(20) $$m\left(A\right)\;=\;\sum_{i=1}^{N}m_{i}\left(A\right).w_{i}$$

**Step 8**: Combine modified data of N time-steps for (N-1) times with DS combination rule using (5) and (6).

## 4. Anti-Disturbing Ability and Transition Property of Proposed Algorithm

The goal of the time-domain fusion is to deal with the conflict between time-domain data. Time domain fusion works as a damper for sudden changes. It also improves accuracy if pieces of evidence are credible. Following example is used to compare results from the literature.

**Example** **2.**  *Assuming that the discriminant frame of a mid-course ballistic target integrated identification system is*Θ={A,B,C}. *Multiple sensors have identified the targeted category at five consecutive moments. The data after the space-domain fusion is provided by multiple sensors at each moment shall be the input data of sequential combination in time-domain, as shown in Table 1.*

From Table 2, it is evident that when the sensors provide the normal data at time-step $T_{1}-T_{3}$, Dempster’s rule, Song-1 method, Song-2 method, Chengkun’s method and the proposed method can make a correct decision at any moment. When the sensors are disturbed at time $T_{4}$, Dempster’s rule has fallen into the trouble of ’one-ballot veto’ paradox and failed to correctly identify m(A). Song-2 method showed slightly better performance against the adversarial information at $T_{4}$ compared to Song-1 but failed to correctly identify m(A). Chenkung’s method showed better robustness against change compared to Song’s method.

**Figure 2:**
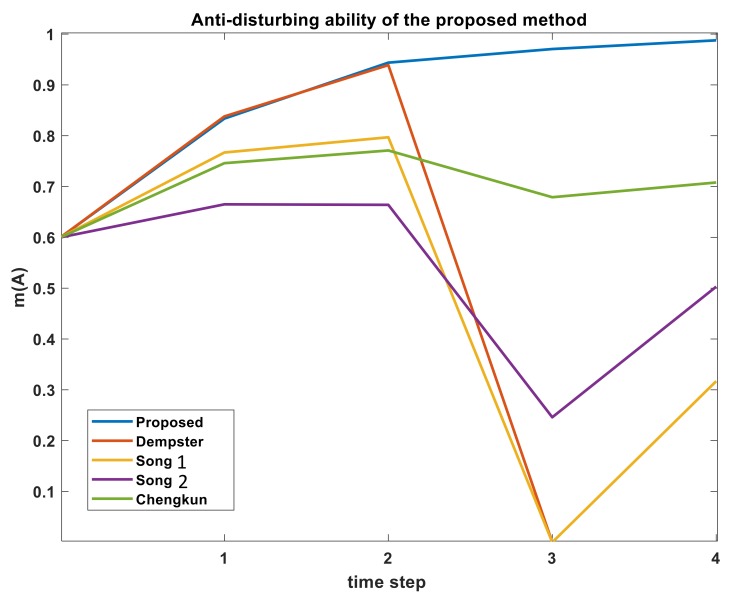
Comparison of anti-disturbing ability of several combination rules for Example 2.

From Figure 2, it is obvious that at time $T_{4}$, the fluctuation of m(A) in the proposed method is non-existent. All the other methods show some extent of fluctuation towards lower m(A) data. But the proposed method penalizes that time-step data and keep improving m(A). This shows that the proposed method can effectively handle the conflicting information of time-domain data and has stronger anti-disturbing ability.

Proposed algorithm puts higher weights on time-steps when data agrees with one another. Also, if at $T_{4}$, a small value of m(A) was used instead of zero (say, m(A) = 0.05), the proposed algorithm would still produce superior fusion results. At $T_{4}$, m(A) value for original Dempster fusion rule would not go to zero but still would be a very small number. Chengkun, Song-1 and Song-2 would produce slightly better fused result with less deviation (or dip) compared to current result but would still contain downward deviation for fused m(A). On the other hand, the proposed method would use the small value of m(A) as a positive reinforcement and would produce higher fused m(A) value compared to the results from Figure 2. When time-step data are transitioning from one BPA to another in time-domain, it would be interesting to see how the proposed algorithm cope with the new time-steps which have higher evidence on a different BPA. Also, how quickly the algorithm can response along the transition. Example 3 is used to test the transition property.

**Example** **3.**  *Assuming that the discriminant frame of a mid-course ballistic target integrated identification system is*Θ={A,B,C}. *Multiple sensors have identified the targeted category at five consecutive moments. The evidence after the spatial fusion of data provided by multi-sensor at each moment shall be the input evidence of sequential combination in time-domain, as shown in Table 3. The target has changed from A to B after time-step $T_{2}$.*

**Figure 3:**
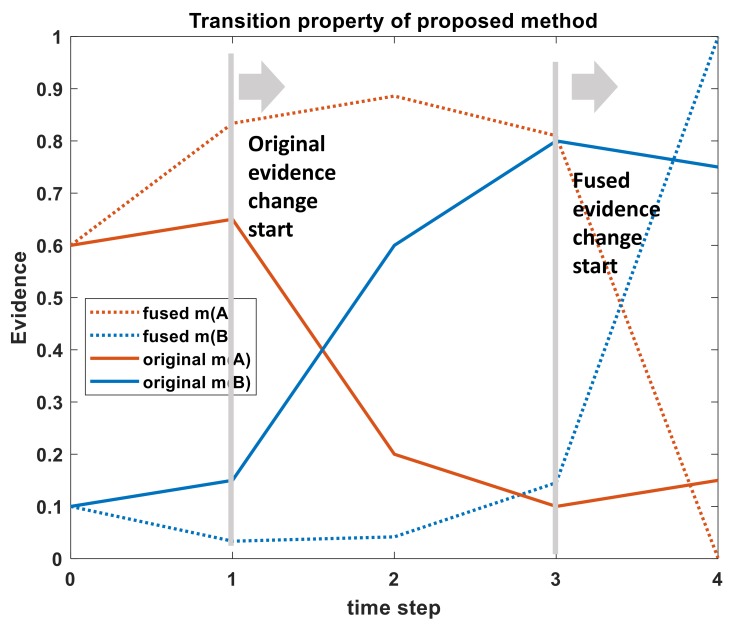
Transition property of the proposed algorithm for Example 3.

At time-domain fusion, another important goal is to make the system robust so that time-step data can transition quickly between one another. As seen from Figure 3, proposed method showed reasonable results for transition between time-step data. Time-step data of m(B) started to rise after $T_{2}$. But Fused m(B) started to rise after $T_{4}$. Based on input evidence, it can be said that the proposed method takes 2 time-steps for time-step data transition which is quite robust for real-time object classification application. As an example, let us say, camera can process video at 30 frames per second (FPS). Each time-step for this case, is time needed to process each frame. Because new time-step data are gathered with each frame. For 30 FPS, each time-step = (1/30) = 0.03 s. If 2 time-steps are needed for proper transition from one fused time-step data to another, it will take (2*0.03) = 0.06 s, which is quite robust for real-time application.

## 5. Modification of BPA for CNN-Based Object Classification under DS Framework

An image convolution is an element-wise multiplication of two matrices followed by a sum. An image is essentially a multidimensional matrix. It has width (number of columns), height (number of rows) and depth (number of channels—for RGB image it is 3). This big matrix (image) is multiplied with a small matrix (kernel) to create the convolution operation. In specific computer vision applications (like edge detection), kernel is hand-defined. As an example, for Sobel edge detection, kernel is a 3-by-3 matrix with zero values at the center column. A machine learning algorithm designed to look at the training images and create these kernels (or filters) to detect specific objects are called convolution neural network. A convolution works by sliding these windows of size 3×3 or 5×5 (n×n sized kernels) over the 3D input feature map (image), stopping at every possible location, and extracting the 3D patch of surrounding features. For real-time object classification, CNN [22] performs better than classic feed-forward neural network or hand-coded feature extraction machine learning systems, because of the following two reasons. First, CNN learns translation invariant properties. It means, features learned by CNN can be applied to anywhere in an image for detection. Secondly, CNN learn spatial hierarchies of patters. Convolution layers at the beginning will learn rudimentary patterns like edges and colors. Higher up layers will learn more complex features.

In this research, a CNN based on VGG16 [23] is fine-tuned to classify three common weeds (Ragweed, Pigweed and Cocklebur) which is commonly found in corn fields. The weed images were taken with a commonly available 16-megapixel digital camera. Maximum input image size used in this study is 150-by-150 pixels. Common Cocklebur (Xanthium strumarium), Redroot Pigweed (Amaranthus retroflexus) and Giant Ragweed (Ambrosia trifida), these three types of commonly found corn weeds are grown in IUPUI Greenhouse to collect images at different stages of growth. Additionally, authors went to actual corn fields during summertime to capture weed images. Each weed category contains roughly 675 images for training and testing purposes. VGG16 (developed by visual geometry group, 16-layers architecture) is a CNN first used multiple small kernel filters instead of single large kernel filters. Final layers of the VGG16 model are retrained and fine-tuned using the weed dataset to classify the 3 types of weeds by exploiting the large amount of visual knowledge already learned from the Imagenet database. A detailed work explaining the effect of CNN architecture on weed classification, real-time processing and effect of noise and motion blur on classification accuracy is under review [24].

Classification report of the CNN classifier is showed in Table 4. In this classification report, accuracy is the most intuitive performance measure which is simply the ratio of correctly predicted observations. Precision looks at the ratio of correct positive observations. Low precision means false positives are high. Recall is a measure of the ability of a classifier to select instances of a certain class from a data set. Low recall means false negatives are high.

**Figure 4:**
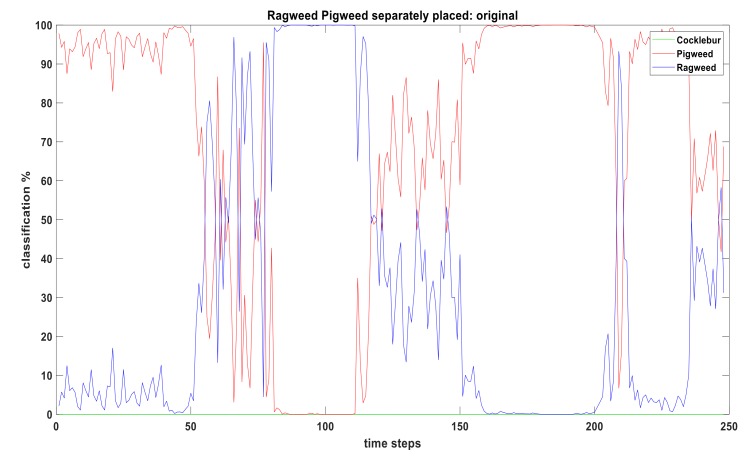
Real-time weed classification from video input using CNN classifier. Classification % is showing CNN output of video feed at each time-step. This CNN output is used as basic probability assignment (BPA) in fusion algorithm.

Figure 4 shows how the CNN classifier performs when a Pigweed plant and a Ragweed plant placed separately (about 1.2 m apart) and video taken from about 0.6 meter above ground. For 0–50 time-steps, CNN is seeing the Ragweed plant base and classifying that as a Pigweed (which is not correct). After 50 time-steps, CNN starts to see the leaves of the Ragweed plant and correct classification starts. Classification percentage of Ragweed starts to rise but the output is noisy and unstable. Between 120–150 time-steps, camera gradually leave behind the Ragweed plant and goes to Pigweed plant (transitional period). After 150 time-steps, correct classification of Pigweed starts. Cocklebur is not present in this video and classification percentage remains zero.

**Figure 5:**
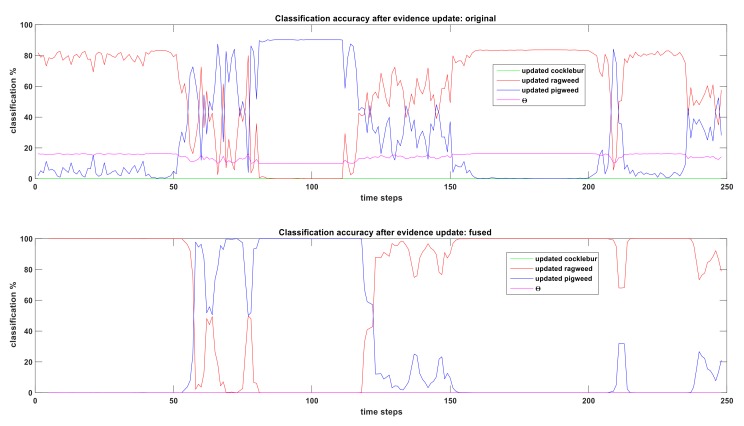
Effect of considering precision and recall on updating BPA on real-time weed classification (top figure). Time-domain fusion of updated BPA for $t_{s}=5$ (bottom figure). Classification % are showing BPA from (21) and (22) (before fusion) (top figure). Classification % showing fused results when BPA from (21) and (22) goes through the proposed fusion algorithm (after fusion) (bottom figure).

The goal is to understand the uncertainties related to CNN classifier and include that within our DS framework. As we have seen, precision and recall are good measures of how well the classifier works at weed classification. The intuition is, the classifier is never 100% certain about any classification even if it shows 100% classification output for any object, because the recall and precision value is not 1.0. Say for an image, the classifier outputs 100% that it is Ragweed. However, among those 100%, only 96% are possibly relevant (Ragweed has 0.96 precision). In addition, among those 96%, only 94% is correctly classified (Ragweed has 0.94 recall). We can include these uncertainties into our BPA using the following equations:

(21) $$m{\left(H_{i}\right)}_{updated}\;=\;Precision_{i}*Recall_{i}*m\left(H_{i}\right)$$

(22) $$m\left(\Theta \right)\;=\;1-\sum_{i=1}^{n}m{\left(H_{i}\right)}_{updated}$$

where Θ={Ragweed,Pigweed,Cocklebur}, is the universal set (Θ in Figure 5) which contains evidence for all three weeds.

Figure 5 shows how incorporating precision and recall into BPA affects real-time weed classification using CNN classifier. Top figure shows the classification results before fusion. As expected, around 20% evidence is placed on Θ (universal set) for all time-steps, which contains evidence for all three weeds. Also, classification accuracy for Ragweed or Pigweed never reaches 100% because remaining evidence is placed in Θ. Effectively, the summation of uncertainties related to CNN classifier is placed into Θ, which is another way of saying that the classifier is not sure about the exact type of weed, so that percentage is placed into Θ because it contains possibility of all three weeds. Bottom figure shows the time-domain sensor fusion (time-step, $t_{s}=5$ used) after evidence update. With updated evidence, time-domain fusion is still successful in filtering noise (reduce sudden changes – smoother curves) from weed classification output and transition from one weed to another. One interesting thing is, Θ value goes to zero which seems counter-intuitive. However, Θ is a set which contains evidence for all three weeds under closed world assumption. During each fusion step (we are using $t_{s}=5$, we have 4 time-domain fusion steps), evidence from the universal set (Θ) is distributed among all three weed pieces of evidence. As a result, Θ value goes down with each fusion step.

## 6. Effect of Number of Time-Steps (Fusion-Time) on Fused Output

**Figure 6:**
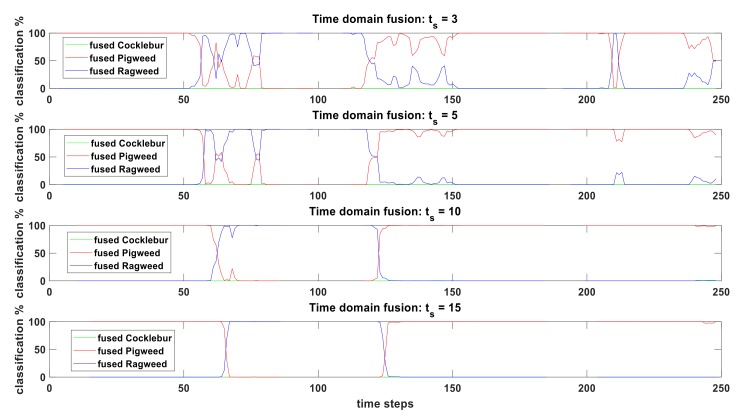
Effect of fusion-time ($t_{s}$) during time-domain sensor fusion on real-time weed classification from video input. Classification % showing fused results when BPA from Figure 4 goes through the proposed fusion algorithm (step 1–step 8).

Number of time-steps considered for time-domain fusion has direct impact on fused output because with increased number of time-steps, higher volume of data is considered for fusion. Figure 6 shows the effect of fusion-time on time-domain sensor fusion. In this figure, fusion-time, $t_{s}=3$ means we consider time-steps ($t_{1},t_{2},t_{3}$) for time-domain sensor fusion. At the next time-step $t_{4}$, we discard $t_{1}$ and consider ($t_{2},t_{3},t_{4}$) for time-domain fusion and the classification output is the fused result of this three time-steps. In addition, this goes on until we reach the end. In this same manner, $t_{s}=5$ means we consider five time-steps ($t_{1},t_{2},t_{3},t_{4},t_{5}$) for time-domain sensor fusion. From Figure 6 it can be seen that fused results for all the time-steps are successful in filtering noise (reduce sudden changes – smoother curves) from weed classification output and transition from one weed to another. In other words, proposed algorithm captures the weed classification dynamics from video feed. Lower $t_{s}$ ($t_{s}=3\;and\;5$) results are more responsive to changes compared to higher $t_{s}$ ($t_{s}=10\;and\;15$) results. Fused weed classification outputs are basically weighted average of the classification values of the fusion-time ($t_{s}$) considered. As a result, if we consider high $t_{s}$ (say $t_{s}=15$), fused output would not change much when at each step, only 1 set of new data is added to a set already containing 15 sets of classification data. This shows that $t_{s}$ is a tuning parameter for time-domain fusion and this can be tuned based on better response (lower $t_{s}$) or better robustness (higher $t_{s}$).

## 7. Conclusions

The proposed 8-step algorithm is applied to time-domain evidence fusion. Proposed algorithm uses the original DS combination rule but improves the fusion results by calculating weights for time-step data. Conflicting time-step data is given lower weights compared to time-step data which agree with one another. Detailed example showed that the proposed method has better combination accuracy in time-domain conflicting information fusion compared to other works from the literature. It also showed better anti-disturbing ability. Transition property of proposed method from one evidence to another proved to be compatible for real-time application. Uncertainties of the CNN-based classifier is included into the fusion algorithm using reconstructed BPA with precision and recall values from the classifier. Evidence-fusion algorithm is tested with real-time video input for weed classification. Number of times-steps ($t_{s}$) considered for time-domain data fusion turned to be an important tuning parameter. Smaller $t_{s}$ values showed fast response in classification output, bigger $t_{s}$ values showed more robustness. Results showed that proposed algorithm can include CNN uncertainties into the evidence-fusion framework and applicable for real-time object classification from video feed.

## Figures and Tables

**Table 1:** Input data of each time-step for Example 2.

Time-Steps	m(A)	m(B)	m(C)
$T_{1}=0$	0.6	0.1	0.3
$T_{2}=1$	0.65	0.15	0.2
$T_{3}=2$	0.6	0.2	0.2
$T_{4}=3$	0	0.85	0.15
$T_{5}=4$	0.55	0.2	0.25

**Table 2:** Data combination results based on different combination methods for Example 2.

Combination Rule	$T_{2}=1$	$T_{3}=2$	$T_{4}=3$	$T_{5}=4$
Dempster [1]	m(A) = 0.838, m(B) = 0.032, m(C) = 0.129	m(A) = 0.939, m(B) = 0.012, m(C) = 0.048	m(A) = 0, m(B) = 0.586, m(C) = 0.413	m(A) = 0, m(B) = 0.531, m(C) = 0.468
Song-1 [10]	m(A) = 0.767, m(B) = 0.076, m(C) = 0.155	m(A) = 0.797, m(B) = 0.091, m(C) = 0.111	m(A) = 0.0, m(B) = 0.843, m(C) = 0.157	m(A) = 0.317, m(B) = 0.458, m(C) = 0.224
Song-2 [9]	m(A) = 0.665, m(B) = 0.077, m(C) = 0.182, m(ϕ) = 0.075	m(A) = 0.664, m(B) = 0.089, m(C) = 0.137, m(ϕ) = 0.109	m(A) = 0.246, m(B) = 0.471, m(C) = 0.135, m(ϕ) = 0.146	m(A) = 0.503, m(B) = 0.27, m(C) = 0.194, m(ϕ) = 0.032
Chengkun [11]	m(A) = 0.746, m(B) = 0.09, m(C) = 0.163	m(A) = 0.771, m(B) = 0.106, m(C) = 0.123	m(A) = 0.679, m(B) = 0.191, m(C) = 0.128	m(A) = 0.708, m(B) = 0.138, m(C) = 0.153
Proposed	m(A) = 0.833, m(B) = 0.033, m(C) = 0.133	m(A) = 0.943, m(B) = 0.017, m(C) = 0.039	m(A) = 0.971, m(B) = 0.007, m(C) = 0.022	m(A) = 0.987, m(B) = 0.002, m(C) = 0.01

**Table 3:** Input data of each time-step for Example 3.

Time-Steps	m(A)	m(B)	m(C)
$T_{1}=0$	0.6	0.1	0.3
$T_{2}=1$	0.65	0.15	0.2
$T_{3}=2$	0.2	0.6	0.2
$T_{4}=3$	0.1	0.8	0.1
$T_{5}=4$	0.15	0.75	0.1

**Table 4:** Classification report of convolution neural network (CNN) classifier.

	Cocklebur	Pigweed	Ragweed
Precision	0.94	0.94	0.96
Recall	1.0	0.89	0.94
Training accuracy	0.99	0.99	0.99
